# Diet quality in cystic fibrosis – associations with patient reported outcome measures and enablers and barriers to eating a healthy diet: A protocol paper for a mixed methods study

**DOI:** 10.12688/hrbopenres.13533.1

**Published:** 2022-05-05

**Authors:** Cian Greaney, Katie Bohan, Sarah Tecklenborg, Brian Casserly, James Green, Pepijn Van de Ven, Katie Robinson, Audrey Tierney

**Affiliations:** 1 School of Allied Health, University of Limerick, Limerick, V94 T9PX, Ireland; 2 Cystic Fibrosis Ireland, 24 Lower Rathmines Rd, Rathmines, Dublin, Ireland; 3 Department of Respiratory Medicine, University Hospital Limerick, Dooradoyle, Limerick, V94 F858, Ireland; 4 Health Research Institute, University of Limerick, Limerick, V94 T9PX, Ireland; 5 Department of Electronic and Computer Engineering, University of Limerick, Limerick, V94 T9PX, Ireland; 6 Health Implementation Science and Technology Cluster, Health Research Institute, University of Limerick, Limerick, V94 T9PX, Ireland; 7 Department of Dietetics, Human Nutrition and Sport, La Trobe University, Melbourne, Victoria, 3086, Australia

**Keywords:** Cystic Fibrosis, Diet Quality, Patient-Reported Outcome Measures, Enablers and Barriers, Healthy Eating.

## Abstract

**Background: **People with cystic fibrosis (PWCF) have increased energy requirements. However, in recent years concerns have emerged regarding the ‘cystic fibrosis (CF) diet’ in terms of reliance on energy-dense, nutrient poor foods, which tend to be higher in saturated fat, sugar, and salt. These foods lack essential nutrients and are aetiologically linked with diet-related chronic diseases. The aim is to explore habitual dietary intakes in PWCF and *(i)* assess adherence to CF dietary guidelines and population specific healthy eating guidelines; *(ii)* derive a diet quality score and the inflammatory potential for the average diet consumed by PWCF and assess associations with patient reported outcome measures; *(iii)* assess drivers for current consumption patterns and enablers and barriers to eating a healthy diet.

**Methods: **The aim is to recruit between 100-180 PWCF. A mixed methods study will be performed. Using three-day food diaries and food frequency questionnaires, aims *(i)* and *(ii) *will be addressed. The Dietary Approaches to Stop Hypertension (DASH) score and Healthy Eating Index-International (HEI-I) will derive diet quality scores. The Dietary Inflammatory Index (DII®) will ascertain inflammatory potential of the diet. Validated questionnaires will be used to report health related quality of life measures. Online focus groups and semi-structured interview with PWCF will address aim *(iii)*.

**Conclusions:** It is timely to revise dietary priorities and targets for CF. However, a greater understanding of what adults with CF currently consume and what they require in terms of nutrition and dietary guidance into the future is needed. In doing so, this research will help to clarify nutrition priorities and simplify the dietary aspects of CF treatment, thereby supporting adherence.

## Introduction

### Background

Cystic Fibrosis (CF) is an autosomal, recessive disorder, arising from defects in the CF transmembrane conductance regulator (CFTR) gene ( Riordan *et al.*, 1989) and affects many aspects of function and daily living for people with CF (PWCF) ( Elborn, 2016; Sawicki *et al.*, 2011). Ireland has the highest incidence of CF worldwide, and approximately 55% of the Irish CF population are in adulthood. This number is anticipated to increase to 65% by 2025 ( Burgel *et al.*, 2015; Farrell, 2008).

It has long been established that PWCF have increased energy requirements due to recurrent pulmonary infections, higher resting energy expenditure from increased work of breathing, and malabsorption ( Kawchak *et al.*, 1996). In the 1960s, a low-fat diet was advocated to manage steatorrhea, which resulted in severe malnutrition and growth failure. In a landmark study, a high-fat diet with aggressive pancreatic enzyme replacement therapy (PERT) resulted in improved growth and survival ( Corey *et al.*, 1988). This has prompted the long-standing clinical practice of prescribing unrestricted high-calorie, high-fat diets with PERT to meet gender specific body mass index (BMI) goals ( Turck *et al.*, 2016). This high energy, high fat ‘CF diet’ contributed to marked improvements in survival and prognosis ( Borowitz *et al.*, 2002; Yankaskas *et al.*, 2004). However, in recent years and with the advancement of genetic modifiers, concerns have emerged in terms of the reliance on energy-dense, nutrient poor foods that tend to be higher in saturated fat, sugar, and salt. In the general population, poor quality diets lack essential nutrients and from a public health perspective are aetiologically linked with diet-related chronic diseases ( WHO & FAO, 2003).

Dietary recommendations in CF are developed based on the best available evidence and expert consensus at that time. Substantial changes in CF treatment and consequent improvements in life expectancy require that guidelines are continuously updated. The European Guidelines advise ad libitum consumption of high-fat foods when weight gain is necessary ( Turck *et al.*, 2016). However, with remarkable improvement in survival, patients with CF may be at risk of developing cardiovascular disease, obesity, and other age-related conditions associated with a high consumption of saturated fat and sugar ( Hanna & Weiner, 2015; Panagopoulou *et al.*, 2014). In paediatric studies, it is reported that children with CF achieve elevated energy requirements through significantly higher intakes of energy-dense, nutrient poor foods ( Sutherland *et al.*, 2018). Children with CF also have micronutrient intakes significantly lower than healthy controls, which has been associated with CF related chronic conditions (e.g., impaired bone health and CF related diabetes) ( Matel & Kerner Jr, 2012; Parkins *et al.*, 2011).

CFTR modulator therapy targets the defective CFTR gene, resulting in improvements in lung function in PWCF that have specific mutations ( Eckford *et al.*, 2012). Ivacaftor (Kalydeco®; Vertex Pharmaceuticals), the first CFTR modulator therapy for individuals with the G551D mutation, along with improving chloride ion transport has been shown to improve duodenal alkalinity ( Rowe *et al.*, 2014). This effect has been documented to continue throughout the distal intestine and resembles alkalinity levels in non-CF individuals ( Gelfond *et al.*, 2013). The normalisation of gastrointestinal (GI) pH is potentially a key feature in the assimilation of food and nutrients ( Rowe *et al.*, 2014). Furthermore, CFTR modulator therapies reduce the risk of lung infections and are associated with improved lung function in PWCF ( Cox *et al.*, 2010; Zemanick *et al.*, 2013). Improved lung function and lower risks of infection can reduce resting energy expenditure in PWCF ( Matel & Milla, 2009; Milla, 2007).

The use of CFTR modulator therapies has resulted in the emergence of overweight and obesity, which will undoubtedly become a more frequent feature in CF. With overweight and obesity in PWCF more than doubling from 14.3% in 2000, to 37% in 2020 ( CFF, 2021), there is an evident upward trend (1.1% increase per annum) towards the general population’s overweight and obesity prevalence rates of 39.0% and 13.0%, respectively ( Statista and WHO). Furthermore, from 2018 to 2020, there was an increase of 7.0% in overweight and obesity cases in PWCF ( CFF, 2019; CFF, 2021), highlighting the acceleration in recent years within this population, coinciding with increases in the use of CFTR modulator therapy ( Solomon & Mallory, 2021). Higher low-density lipoprotein (LDL) cholesterol and triglyceride levels have been found in PWCF who are overweight, which may be influenced by diet related factors ( Loria *et al.*, 2014). A recent review on the historical perspective of dietary intake studies in children with CF stated that ‘the nutrition dogma has to be extended from nutrition for growth and survival to nutrition for health and well-being’ ( Sutherland *et al.*, 2017). This call for practice change needs also to extend to adults with CF.

In the general population, an emphasis on diet quality above individual macronutrients is the basis of dietary recommendations to reduce chronic disease risk ( You, 2015). Certain dietary patterns are associated with beneficial health outcomes including the Mediterranean and Dietary Approaches to Stop Hypertension (DASH) diets ( Bach-Faig *et al.*, 2011; Wengreen *et al.*, 2013). Many diet quality index tools have been developed and validated, albeit, not in the CF population. The Diet Quality Index – International (DQI-I) was constructed to provide a global tool for monitoring healthfulness of diet and for exploring aspects of diet quality. The DASH score has also been previously used within the Irish population and is based on the intake of nine food groups: wholegrains, fruit, vegetables, legumes, low-fat dairy foods, red processed meat, sweetened snacks and beverages, salty snacks, and sodium consumption ( Harrington *et al.*, 2011). Calculation of the Dietary Inflammatory Index (DII®) is another novel method for assessing the theoretical inflammatory impact of a diet ( Shivappa *et al.*, 2014). Whilst attaining disease specific nutrient targets in CF is important, a composite measure of representing whole of diet quality is preferred. However, evidence-based research is lacking to guide CF-specific nutritional recommendations or to promote an optimal dietary pattern.

Few studies have investigated perceptions and experiences of diet with adult PWCF. A recent UK study of adult PWCF (n= 9) uncovered participants had each developed a plan to regulate personal nutritional needs, which included eating well and choosing foods of higher caloric value, motivated by the opinion that better weight will certify better health ( Barrett *et al.*, 2021). Furthermore, another UK study involving adult PWCF (n= 10) sharing childhood experiences of diet, discovered while some participants embraced a high calorie, high fat diet, others pursued a diet with balance that would benefit long-term health ( Cave & Milnes, 2020).

More research is needed to evaluate if a diet that meets the increased energy requirements of PWCF while maintaining the nationally recommended proportions of food groups is feasible and implementable in Ireland. Thoughts on healthy eating and willingness to change habitual dietary patterns to be more healthful whilst still maintaining overall energy targets is required from the patient population prior to any intervention or practice change being employed. Compliance with the ‘CF diet’ is generally poor with estimated adherence rates between 40-55% ( O'Donohoe & Fullen, 2014). With social, behavioural, and physical factors influencing the ability of individuals with CF to follow dietary recommendations ( Brown *et al.*, 2018), exploring the role that these factors play in the consumption of typical dietary patterns and what strategies would be most effective for improving diet quality will determine next steps in research programme planning. Within this, there is growing evidence that patient-reported outcome measures (PROMs) can serve as valuable indicators for the benefit of interventions and the impact on health-related quality of life (HRQoL), as they represent direct measures of how a patient feels and functions ( Solem *et al.*, 2016).

### Aims and objectives

The overall aim of the study is to provide a comprehensive evaluation of habitual dietary intakes, dietary patterns, and the overall diet quality of PWCF living in Ireland and to investigate the interrelationships between diet quality and patient reported outcome measures. Through online focus groups (OFGs), the experiences and views on nutrition and diet in this cohort, including drivers of food and dietary choices, and investigating enablers and barriers for dietary changes will also be explored.

## Methods

### Study design

This study is an observational, cross-sectional study with a mixed methods approach. Participant eligibility will be confirmed using a screening questionnaire. Quantitative data will be collected using a demographic and self-reported health questionnaire, three-day food diary, semiquantitative food frequency questionnaire (FFQ), the Cystic Fibrosis Questionnaire-Revised ( CFQ-R), EuroQol 5-dimension 5-level questionnaire ( EQ-5D-5L), Patient Assessment of Upper Gastrointestinal Symptoms questionnaire (PAGI-SYM) ( Rentz *et al.*, 2004; Revicki *et al.*, 2004; Wyrwich *et al.*, 2010), Patient Assessment of Constipation Symptoms questionnaire (PAC-SYM) ( Frank *et al.*, 1999) and an originally designed post-participation questionnaire (PPQ). Qualitative data will be collected via OFGs and semi-structured interviews, designed to develop discussions amongst participants on experiences and beliefs related to diet, and the impact CF and CFTR modulator therapy plays. The STROBE reporting guidelines will be adhered to for this observational study ( Cuschieri, 2019; Vandenbroucke *et al.*, 2007). The three-day food diary, PPQ, focus group script and screening form can be found as *Extended data* ( Greaney *et al*., 2022).

### Study population

***Participant recruitment***

The aim is to recruit 100-180 PWCF for this observational study using a convenient sampling method ( Etikan *et al.*, 2016). Recruitment will be carried out through Cystic Fibrosis Ireland (CFI) membership forums, CF hospital clinics, and online platforms (e.g., Instagram; Facebook; Twitter; LinkedIn). The gold standard method for investigating sample size sufficiency in qualitative research is a saturation point, and thus, will be used to justify the sample size of the OFGs and semi-structured questionnaires. Data collection and analysis will be conducted concurrently and when saturation has been achieved, described in thematic analysis as when no new codes are identified in participants accounts, recruitment will cease ( Vasileiou *et al*., 2018). From previous qualitative studies, saturation commonly occurs near 30 participants, which set the target sample size for this study. The goal is to run four to five OFGs with six participants in each group (total n= 24-30).

***Participant eligibility***

*Inclusion criteria*: To be included in this study, participants must be adults ≥18 years of age with a diagnosis of CF, living in Ireland. *Exclusion criteria:* Participants will be excluded from the study if they are receiving enteral nutrition, following a prescription diet for another medical condition (e.g., coeliac disease, are pregnant) or are post-transplant. Adults must be on a stable medical regimen for at least four weeks prior to commencing the study with no recent pulmonary exacerbations involving the administration of oral or intravenous antibiotics or glucocorticoids.

### Data collection

Both quantitative and qualitative data will be collected as part of the study. Participants will be asked to complete a demographic and self-reported health questionnaire offered in online and paper-based versions. Patient-reported clinical and demographic data will be collected to describe the interview sample, including age, sex, most recent BMI, and lung function (forced expiratory volume in 1 second (FEV _1_%)); CF-related comorbidity status (e.g., presence of CF-related diabetes, liver disease) and medications including the use of genetic modulator agents.

As part of the quantitative dietary assessment, participants will be advised to complete an estimated three-day food diary, either paper-based or using the smartphone application Libro from the Dietary Analysis Software Nutritics, Version 5.7 Research Edition (Nutritics, RRID:SCR_022154) (Nutritics Ltd.: Dublin, Ireland). Participants will also complete a semi-quantitative FFQ. The FFQ is adapted from the European Prospective Investigation of Cancer ( EPIC) FFQ ( Harrington *et al.*, 2008), validated for use in the Irish general population ( Harrington, 1997).

The DQI-I and the DASH score will be used to derive diet quality scores for each participant ( Fung *et al.*, 2008; Harrington *et al.*, 2011). DII® has been developed to collate all the individual food or nutrient intake components that have a known effect on markers of inflammation ( Shivappa *et al.*, 2014). Based on the intake of each of these components in the index, an individual’s diet can be assessed for its pro- or anti-inflammatory potential.

PROMs used in the study include the following: CFQ-R, a disease-specific, validated tool that encompasses general domains of HRQoL as well as domains specific to CF ( FDA, 2009; Henry *et al.*, 2003; Modi & Quittner, 2003; Quittner *et al.*, 2005). The universal evaluation tool, EQ-5D-5L, produces a single index measurement of health status and elicits means of comparability for treatments of differing medical conditions ( Eidt-Koch *et al.*, 2009; Rabin & Charro, 2001; Solem *et al.*, 2016). The 20-item PAGI-SYM and the 12-item PAC-SYM are validated instruments for monitoring abdominal symptoms ( Rentz *et al.*, 2004; Revicki *et al.*, 2004; Slappendel *et al.*, 2006).

Participants will be invited to take part in an OFG, or semi-structured interview (max. one hour) facilitated by a trained, experienced dietitian, conducted over Microsoft Teams. OFGs will be conducted in groups of four to six. However, if participants prefer to be interviewed face-to-face or by telephone this will be facilitated. OFGs and interviews will be video, and audio recorded for later transcription and analysis. The readability of OFG questions will be measured using the Flesch-Kincaid Readability Test, a validated test that produces a value based on readability level and education level ( Flesch, 2007; Heydari, 2012). The questions designed for the OFGs/interviews will cover three broad topics: views on diet advised/promoted for PWCF; current consumption patterns; enablers and barriers to eating a healthier diet. Once all topics have been discussed, participants will be invited to complete the PPQ administered in the video call chatroom or via email, to facilitate engagement. The PPQ was developed on Microsoft Forms and will be used to gather quantitative information to reinforce beliefs of PWCF articulated in the OFGs and semi-structured interviews.

### Data management

The primary researcher will be responsible for the storage of hard copies of signed consent forms and questionnaires, which will be kept in secure filing cabinets in line with the 2018 Data Protection Act ( *Data Protection Act*, 2018). Electronic data will be saved on a cloud-based password-protected database. Data will be anonymised prior to data extraction. The Electronic Data Capture system (EDC) Castor, Version 1.6 (Castor – Electronic Data Capture System RRID:SCR_022150) (Ciwit B.V., The Netherlands) a cloud-based, clinical data management platform will be used to store all quantitative data collected. Personal data will only be available by key researchers involved in the study and will be anonymised before being entered into a dataset to be analysed. After the dataset has been analysed and the report/paper written, the dataset will be preserved for seven years before being destroyed. Any participants who are interested in the results of the research will be flagged and both individual and overall results will be disseminated after study completion.

### Data processing and analysis

All statistical analyses will be conducted in SPSS® Statistics for Windows, Version 28 (IBM SPSS Statistics, RRID:SCR_016479) (IBM Corp., Released 2019). Data will be presented as means ± standard deviations (SD), medians and interquartile ranges (IQR), and frequencies (n and %) as appropriate.

Macronutrient and micronutrient intakes will be quantified as absolute intake and energy-adjusted intake (amount per 1000kcals). Appropriate descriptive statistics will be used to describe the baseline characteristics of the cohort. Distribution of data will be tested using the Kolmogorov Smirnov test of normality, where a significance of *p* > 0.05 will identify data as being normally distributed. P-values will be considered significant if the p-value shows a result of *p* < 0.05. Independent t-tests (or Mann Whitney for non-parametric variables) will be used to compare continuous variables between participant grouping of two categories (e.g., sex differences). One-way analysis of variance (ANOVA) (or Kruskal-Wallis for non-parametric variables) tests will be applied to compare continuous variables between participant groupings of three or more categories (e.g., age categories) and Post Hoc Tukey tests will be performed to assess differences between specific categories. Pearson's Chi-square will be used to test differences between categorical variables. Total diet quality scores and scores for each component of the score will be calculated and compared between demographic groups using appropriate statistical tests as previously described. Associations between diet quality, participant demographic variables and patient reported outcome measures will be investigated. Pearson or Spearman rho correlations will be used to investigate associations between diet quality scores and DII® scores. Multiple (linear) regression models will be performed to assess what variables most strongly predict increased diet quality scores and an increased dietary inflammatory index. Dietary patterns will be identified from the FFQ data by principal components analysis.

A qualitative inductive thematic analysis method described by Braun and Clarke (2006) will be completed using NVivo ^®^ (NVivo, RRID:SCR_014802) (QSR International Pty Ltd.), a qualitative data analysis computer software package, to analyse data collected from the OFGs and semi-structured interviews. Previous research has found this tool useful in organising large amounts of data ( Castleberry, 2014; Castleberry & Nolen, 2018). The approach to thematic analysis by Braun and Clarke (2006) consists of six-phases: 1) data immersion; 2) generating codes; 3) generating themes; 4) reviewing potential themes; 5) defining and naming themes; 6) producing a report.

Transcripts from each OFG and interview will initially be read several times to immerse the researcher in the data, a preparation phase prior to further analysis to familiarise the researcher with descriptions and language of participants ( Braun & Clarke, 2006). Following this, the researcher will code the data inductively in NVivo ^®^ and a second researcher will also code the transcripts. After refining of codes is complete, subsequential themes will be actively generated supported by discussion with the wider research team.

### Ethical approval and informed consent

This study has received funding by the Health Research Board (HRB) and Health Research Charities Ireland (HRCI) grant initiative with CFI. Ethical approval has been granted by University of Limerick Education and Health Sciences, Research and Ethics Committee, University Hospital Limerick (Ref: 090/2021; Date of Approval: 22 ^nd^ September 2021) and Cork University Hospital (Ref: ECM 4 (o) 11/01/2022& ECM 3 (bb) 22/02/2022; Date of approval: 26 ^th^ January 2022), and University Hospital Galway Research and Ethics Committee (Ref: C.A. 2709; Date of approval: 10 ^th^ March 2022) for all aspects of the study. Once PWCF volunteer to participate in the study a screening questionnaire will be used to determine participant’s eligibility. Once completed, all eligible participants will be provided with a participant information form and consent form (PICF) to read and sign prior to taking part in the study. Depending on participant preference, all questionnaires will be offered in either an online or paper-based version. All participants will be informed that participation is voluntary, and they can withdraw at any point during the study. Once written consent is obtained, participants are enrolled in the research study. Verbal consent will be requested for recording of the OFGs or semi-structured interviews during each introduction. This study is in compliance with the Declaration of Helsinki ( WMA, 2014).

### Data dissemination

A report on the findings will be prepared and presented to the funding body HRB, CFI, and to national and international project collaborators. Findings will also be presented at the CFI annual conference. Manuscripts will be prepared for publication and submitted to relevant journals. Abstracts will be submitted to conferences.

### Study status

Currently the research team are undergoing recruitment of participants via CFI membership forums, CF hospital clinics and online platforms.

## Discussion

First recommended in the 1960s, the long-standing clinical practice of prescribing an unrestricted, high calorie and high fat diet with PERT to improve growth and survival rates in PWCF is still in place today, despite the development and known effects of effective treatments like CFTR modulator therapy ( McDonald *et al.*, 2021). With heightened concerns in recent years over the ‘CF diet’, which tends to be high in saturated fat, sugar and salt, aetiologically linked with diet-related chronic disease in non-CF populations, previous research has expressed that the established nutritional opinion of eating for growth and survival in PWCF, must now extend to nutrition for health and wellbeing ( McDonald *et al.*, 2021; Sutherland *et al.*, 2017).

The accelerated growth of overweight and obesity in PWCF observed in recent years (3.5% increase per annum - 2018–2020; 1.1% increase per annum - 2000–2020), has coincided with an increased use of CFTR modulator therapy ( CFF, 2019; CFF, 2021; Solomon & Mallory, 2021). One of the potential impacts of CFTR modulator therapies is that the aetiological link between diet-related chronic diseases and a high-calorie, high fat diet observed in the general population, may also become prevalent in the CF population who are treated with CFTR modulator therapies that are more likely to result in lower energy expenditure and improved absorption of food and nutrients. Therefore, an alternative diet to the ‘CF diet’, with links to positive health outcomes (e.g., Mediterranean ( Bach-Faig *et al.*, 2011)/DASH diets ( Wengreen *et al.*, 2013)/adherence to national dietary guidelines) may be important to prevent the development of diet-related chronic disease.

With compliance to the ‘CF diet’ generally poor ( O'Donohoe & Fullen, 2014), the effects of social, behavioural, and physical factors on dietary recommendation adherence are important to explore. PWCF face many nutritional challenges daily, aiming to achieve a normal body composition and managing comorbidities. While most of the nutrition and diet related studies in this area document nutrient intakes, there is a lack of data on dietary patterns and diet quality. In addition, to the researcher’s knowledge, few studies have addressed the drivers and barriers to eating a healthy diet for PWCF, the role that disease, socioeconomic and other factors play in the consumption of typical dietary patterns; models to promote dietary variety and quality; promote a healthy weight status and address metabolic health concerns and chronic disease risk. This study will address these gaps.

This research programme has been designed to have a strong translational focus with recruitment conducted through the patient organisation CFI and CF clinics across Ireland. It has the potential to change current dietary CF practices internationally, with outcomes translated to the clinical setting to influence models of care for dietetic services in CF centres in Ireland and further afield, possibly providing a basis for future interventions. Individuals taking part in the OFGs may also benefit in psychosocial terms from sharing their experiences with other people in a friendly and supportive environment. This may potentially translate to a feeling of empowerment amongst PWCF participating in the study, as it allows individuals to voice their opinions in a constructive environment.

A possible study limitation could be the emergence of social desirability bias. Social desirability bias refers to a participant’s susceptibility to respond in a form they deem to be more socially acceptable, as opposed to providing true beliefs or feelings, and is often present in interviews/focus groups on socially sensitive issues (e.g., personal issues; environment; smoking; religion). However, the use of a well-trained facilitator can prevent this form of bias occurring ( Grimm, 2010). Another limitation will be that OFG participants will not be stratified into specific groups (e.g., age category, gender, socio-economic status). This is limiting as stratification of participants frequently results in higher comparability levels, potentially producing richer data ( Morgan, 1996). Nonetheless, there will be an allowance for this limitation to recruit the optimal number of participants by placing PWCF in OFGs based on when best suits them to participate, and who first becomes available for the study. A final limitation will be that dietary evaluation will be conducted via an estimated three-day food diary and FFQ. An estimated food diary relies on portion size estimation, in comparison to the gold standard method for assessing dietary intake, a weighed food diary, which provides more precise measurements of portions ( Ortega *et al.*, 2015). Nonetheless, the three-day estimated food diary method will be utilised to reduce burden on participants. Food records less than seven days long have also been observed to reap more accurate results, reducing the risk of information bias, thus maintaining validity and reliability ( Shim *et al.*, 2014). The FFQ chosen is validated for use in the Irish population, justifying its inclusion ( Harrington, 1997).

In summary, the connection between the ‘CF diet’ and overweight, obesity and other diet-related chronic diseases has become a cause for concern, identifying the potential need for change from eating for growth and survival to eating for health and wellbeing in PWCF. The advancements in gene therapy and the subsequent introduction of CFTR modulator therapy to treat PWCF, has resulted in increased rates of overweight and obesity, highlighting it is now prudent to establish an alternative diet for PWCF. This study is intended to be the first that investigates dietary patterns and diet quality in adult PWCF, addressing the drivers and barriers to eating a healthy diet, the role that disease, socioeconomic and other factors play in the consumption of typical dietary patterns, the models to promote dietary variety and quality, promote a healthy weight status and address metabolic health concerns and chronic disease risk. This research has the potential to change CF dietary practice worldwide, influencing models of care for dietetics services and future interventions to improve the quality of life of PWCF.

## Data availability

### Underlying data

No data are associated with this article.

### Extended data

Figshare: Diet quality in cystic fibrosis study. https://doi.org/10.6084/m9.figshare.c.5964891. ( Greaney *et al*., 2022).

This project contains the following extended data:

- 3-day Food Diary
- Post Participation Questionnaire
- Focus Group Script
- Screening Form - Diet in CF

Data are available under the terms of the Creative Commons Zero "No rights reserved" data waiver (CC0 1.0 Public domain dedication).
