# Supplementary material for: Diet quality in cystic fibrosis – associations with patient reported outcome measures and enablers and barriers to eating a healthy diet: A protocol paper for a mixed methods study
Source: HRB Open Res. 2025 Sep 17;5:33. Originally published 2022 May 5. [Version 2] doi: 10.12688/hrbopenres.13533.2 (PMC9428499; doi:10.12688/hrbopenres.13533.2)
Supplement: Peer review history

# Reviewer report: Reviewer response for version 2

**DOI:** 10.21956/hrbopenres.15680.r49981

**Published:** 2025-09-23

**Authors:** Catherine M. McDonald

**Affiliations:** 1 Department of Clinical Nutrition, Primary Children's Hospital, Salt Lake City, UT, USA

No additional comments. Approved.

Is the study design appropriate for the research question?

Yes

Is the rationale for, and objectives of, the study clearly described?

Yes

Are sufficient details of the methods provided to allow replication by others?

Yes

Are the datasets clearly presented in a useable and accessible format?

Not applicable

Reviewer Expertise:

CF nutrition (pediatric)

I confirm that I have read this submission and believe that I have an appropriate level of expertise to confirm that it is of an acceptable scientific standard.

# Reviewer report: Reviewer response for version 1

**DOI:** 10.21956/hrbopenres.14769.r32471

**Published:** 2022-08-30

**Authors:** Megan Gabel

**Affiliations:** 1 Department of Pediatrics, School of Medicine, University of Rochester, Rochester, NY, USA

Diet quality in people with Cystic Fibrosis (CF) is emerging as an important topic with very little data to inform updated recommendations regarding best practices. The increasing rates of overweight and obesity in the CF population have highlighted the need to revisit nutritional recommendations. This study is an important step in understanding what the dietary intake of people with CF is currently, and will help to identify barriers to eating a high-quality diet. While high-calorie diets may remain important for some people with CF, understanding how we can achieve this without compromising diet quality will be helpful.  

 Regardless of weight status, the focus on nutrient poor foods has the potential for health complications over time. I agree with the other reviewer that assessing food insecurity would be a valuable component of this study.

 Including the OFGs will be an important way to identify both barriers and potential opportunities to better partner with people with CF regarding dietary recommendations.

Is the study design appropriate for the research question?

Yes

Is the rationale for, and objectives of, the study clearly described?

Yes

Are sufficient details of the methods provided to allow replication by others?

Yes

Are the datasets clearly presented in a useable and accessible format?

Partly

Reviewer Expertise:

Pediatric Gastroenterology and Nutrition

I confirm that I have read this submission and believe that I have an appropriate level of expertise to confirm that it is of an acceptable scientific standard.

# Response

**Published:** 2022-09-29

**Authors:** Cian Greaney

**Affiliations:** University of Limerick, Ireland

Dear Reviewers,

 Thank you for taking the time to review and approve our study protocol. This is a response to the questions raised in relation to our research.

**Q1. BMI is obtained, but are reported weight and height gathered to double check the estimated BMI?  Date of last CF clinic visit and or hospitalization? Did self-reported measurements come from the last CF clinic visit or home measurements, or something else?**

 We require participants to provide their height and weight to estimate BMI, the date of their last hospital admission, length of stay, and number of admissions in the past year, as part of the self-reported health and demographics questionnaire. FEV _1_% measurements are advised to come from participant’s last outpatient clinic appointment where possible. All other measurements (i.e., height; weight; age) are self-reported by the participants with Cystic Fibrosis (CF) and not required to come from the last clinic appointment.

**Q2. Are there food security questions such as the Hunger Vital Sign, a validated 2 question screening tool?  (1. Within the past 12 months, we worried whether our food would run out before we got money to buy more. 2. Within the past 12 months the food we bought just didn't last and we didn't have money to buy more.)?**

 Factors impacting food security are gathered as part of the self-reported health and demographics questionnaire. These included: current living situation; locality; description of where they currently live; employment status; education level. The CFQ-R questionnaire explores how CF impacts some of these social factors including school, work, and daily tasks, while the EQ-5D-5L health questionnaire assesses health related to mobility and self-care, other influences on food security. Furthermore, within online focus groups and semi-structured interviews, interviewers ask whether finances, time or accessibility to food has ever impacted food choice (see extended data: Focus Group Script). In the post participation questionnaire, these same factors are assessed further, requiring participants to give a rating on a scale of 0-10 on how much they impact food choice (see extended data: Post Participation Questionnaire). Thus, we feel food security is effectively assessed in our research without the introduction of the Hunger Vital Sign screening tool.

**Q3. Are the PWCF recruited for this study being given instructions on how to estimate food beverage portions or how to accurately describe the details of food/beverage consumed?  For example, is the hamburger recorded on the food log just plain cooked ground beef, a fast-food kiddie burger, or a deluxe double meat, double cheeseburger with bacon and extra mayo on an artisan bun? In addition to food and beverage intake, will participants report enzyme use with each meal and snack?  This could impact absorption and would be important information. Is salt added? if so, how much? Is water consumption included in the dietary intake records?**

 Participants are given appropriate instructions on how to correctly estimate food/beverage portions and to accurately describe the details of food/beverage consumed. A sample food diary is provided to demonstrate how food and drink should ideally be recorded. Participants are also required to report enzyme use with each meal, salt and fluids as outlined in the 3-day food diary (see extended data: 3-day Food Diary).

 I hope I have addressed all your questions. Feel free to contact me about the study at any time (contact details provided).

 Kind regards,

 Cian Greaney

# Reviewer report: Reviewer response for version 1

**DOI:** 10.21956/hrbopenres.14769.r31993

**Published:** 2022-05-27

**Authors:** Catherine M. McDonald

**Affiliations:** 1 Department of Clinical Nutrition, Primary Children's Hospital, Salt Lake City, UT, USA

As noted by the authors, there is a dearth of information regarding the dietary habits of adults with cystic fibrosis. The dietary quality and variety in pediatric people with cystic fibrosis (PWCF) has been shown to be less than optimal when compared to recommendations for the general population and not conducive to long-term good health. The research project described in this article will give insight into the current state of nutrient intake for PWCF and provide direction for improving the nutrition therapy offered to PWCF.  

 The following items are not specified in the article but may be in the research protocol. In the data collection phase, additional useful information would include subjects' genotype, history of meconium ileus, or intestinal surgery.  Also, BMI is obtained, but are reported weight and height gathered to double check the estimated BMI?  Date of last CF clinic visit and or hospitalization? Did self reported measurements come from the last CF clinic visit or home measurements, or something else? 

 Are there food security questions such as the Hunger Vital Sign, a validated 2 question screening tool?  (1. Within the past 12 months, we worried whether our food would run out before we got money to buy more. 2. Within the past 12 months the food we bought just didn't last and we didn't have money to buy more.)? 

 Food insecurity is important to identify because it impacts both the quantity and the quality of the food available to the individual. Food insecurity and chronic disease can be cyclical, with one problem exacerbating and perpetuating the other.  Individuals with chronic diseases such as cystic fibrosis and other factors such as living in a household with children, headed by a single parent, or a household with income near or below poverty levels may be more vulnerable to experiencing food insecurity. ^1^^,^^2^^,^^3^.

  The following questions come from my experience as a clinical dietitian  who works with PWCF's diet records: 

- Are the PWCF recruited for this study being given instructions on how to estimate food /beverage portions or how to accurately describe the details of food/beverage consumed?  For example is the hamburger recorded on the food log just plain cooked ground beef, a fast food kiddie burger, or a deluxe double meat, double cheeseburger with bacon and extra mayo on an artisan bun?  
- In addition to food and beverage intake, will participants report enzyme use with each meal and snack?  This could impact absorption and would be important information.
- Is salt added? If so, how much? 
- Is water consumption included in the dietary intake records? 

  I look forward to seeing the results of this study.

Is the study design appropriate for the research question?

Yes

Is the rationale for, and objectives of, the study clearly described?

Yes

Are sufficient details of the methods provided to allow replication by others?

Yes

Are the datasets clearly presented in a useable and accessible format?

Not applicable

Reviewer Expertise:

CF nutrition (pediatric)

I confirm that I have read this submission and believe that I have an appropriate level of expertise to confirm that it is of an acceptable scientific standard.

## References

[1] Cystic Fibrosis Food Security Screening and InterventionQuality Improvement Change Package. *Cystic Fibrosis Foundation* .2022; Reference source

[2] Food insecurity and health: Resource toolkit. A guide for screening individual and referring to resources for help, designed for healthcare and non-healthcare professionals. *Humana Feeding America* .2022; Reference source

[3] : Evaluation of Food Insecurity in Adults and Children With Cystic Fibrosis: Community Case Study. *Front Public Health* .2018;6: 10.3389/fpubh.2018.00348 348 10.3389/fpubh.2018.00348 30534549 PMC6275191
